# Lipid-Modified Azurin of *Neisseria gonorrhoeae* Is Not Surface Exposed and Does Not Interact With the Nitrite Reductase AniA

**DOI:** 10.3389/fmicb.2018.02915

**Published:** 2018-11-27

**Authors:** Benjamin I. Baarda, Ryszard A. Zielke, Ann E. Jerse, Aleksandra E. Sikora

**Affiliations:** ^1^Department of Pharmaceutical Sciences, College of Pharmacy, Oregon State University, Corvallis, OR, United States; ^2^Department of Microbiology and Immunology, F. Edward Hebert School of Medicine, Uniformed Services University of the Health Sciences, Bethesda, MD, United States; ^3^Vaccine and Gene Therapy Institute, Oregon Health & Science University, Beaverton, OR, United States

**Keywords:** *Neisseria gonorrhoeae*, Laz, cell envelope, anaerobic respiration, vaccine, mouse model, *in vivo* cross-linking

## Abstract

Lipid-modified cupredoxin azurin (Laz) is involved in electron transport in *Neisseria* and proposed to act as an electron donor to the surface-displayed nitrite reductase AniA. We identified Laz in *Neisseria gonorrhoeae* cell envelopes and naturally elaborated membrane vesicles in proteomic investigations focused on discovering new vaccine and therapeutic targets for this increasingly difficult to treat pathogen. Its surface exposure in *N. meningitidis* suggested Laz could be a vaccine candidate for *N. gonorrhoeae*. Here we characterized the localization, expression, and role of Laz within the gonococcal cell envelope and challenged the hypothesis that Laz and AniA interact. While we demonstrate that Laz indeed shows some good features of a vaccine antigen, such as stable expression, high conservation, and ability to elicit antibodies that cross-react with a diverse panel of *Neisseria*, it is not a surface-displayed lipoprotein in the gonococcus. This discovery eliminates Laz as a gonorrhea vaccine candidate, further highlighting the necessity of examining homologous protein localization between closely related species. Absence of Laz slightly altered cell envelope integrity but was not associated with growth defects *in vitro*, including during anoxia, implicating the presence of other electron pathways to AniA. To further dissect the implied AniA-Laz interaction, we utilized biolayer interferometry and optimized and executed chemical cross-linking coupled with immunoblotting to covalently link interacting protein partners in living gonococci. This method, applied for the first time in *N. gonorrhoeae* research to interrogate protein complexes, was validated by the appearance of the trimer form of AniA, as well as by increased formation of the β-barrel assembly machinery complex, in the presence of cross-linker. We conclude that Laz is not an electron donor to AniA based on their distinct subcellular localization, discordant expression during infection of the female mouse lower genital tract, and lack of interaction *in vivo* and *in vitro.*

## Introduction

Azurins are a class of small copper-containing proteins with a characteristic strong spectroscopic absorbance at 600 nm (Ainscough et al., 1987; Nobrega et al., 2016). Originally described in *Pseudomonas aeruginosa* as “*Pseudomonas* blue protein” (Horio, 1958a,b), azurin was subsequently named according to pigment nomenclature after biochemical characterization in *Bordetella* species (Sutherland and Wilkinson, 1963). Azurin demonstrates a strong redox potential (Sutherland and Wilkinson, 1963), which allows it to function as an electron carrier for periplasmic or membrane-bound components of the electron transport chain, especially between cytochrome *c*-551 and cytochrome oxidase (Holwerda et al., 1976; Farver and Pecht, 1991). This electron shuttling ability enables azurin to protect *P. aeruginosa* from oxidative stress *in vitro* (Vijgenboom et al., 1997).

Azurin proteins are not universally distributed among bacteria, nor are they present in all pathogenic bacteria (Sutherland and Wilkinson, 1963). However, they are found in *Neisseria* species, including pathogenic *N. meningitidis* and *N. gonorrhoeae*, as well as commensal *N. lactamica, N. flava*, and *N. perflava* (Woods et al., 1989). In *Neisseria*, unlike in other bacteria, azurin contains a lipoprotein signal peptide, including an invariant cysteine residue that is recognized by signal peptidase II and lipid modified to tether it to the membrane (Woods et al., 1989; Nakayama et al., 2012). Similar to *P. aeruginosa*, lipid-modified azurin (Laz) protected *N. meningitidis* and *N. gonorrhoeae* against hydrogen peroxide-induced oxidative stress (Wu et al., 2005). However, unlike in *P. aeruginosa*, paraquat- or xanthine/xanthine oxidase-generated superoxide had no effect on Δ*laz* mutants in either *Neisseria* species (Wu et al., 2005). Additionally, Laz appears to be surface exposed in *N. meningitidis*, as illustrated by the sensitivity of a non-encapsulated strain to the bactericidal effect of anti-Laz antiserum (Deeudom et al., 2015).

**FIGURE 1:**
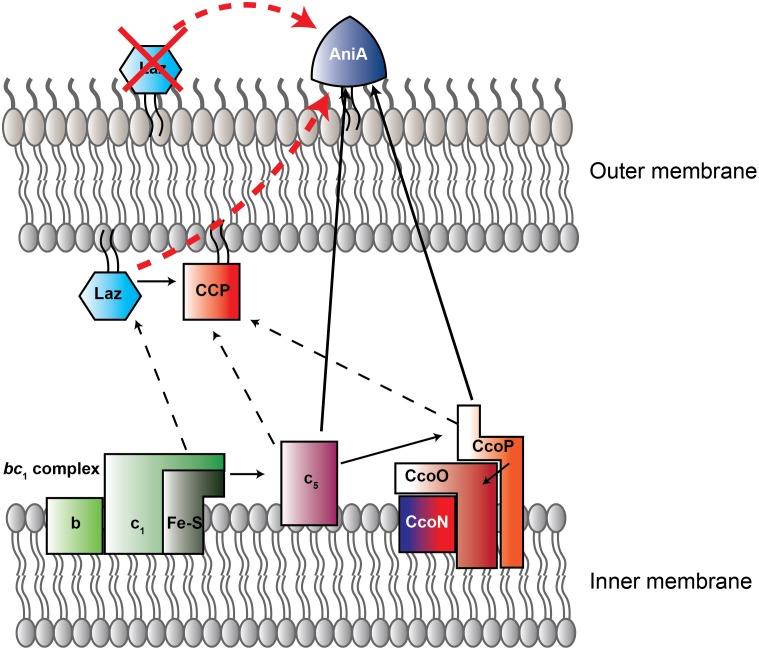
New model of electron transport chain in *Neisseria gonorrhoeae*. Schematic representation of electron transfer chain in *N. gonorrhoeae* built on current and previous studies (Hopper et al., 2013; Nobrega et al., 2016). Arrows indicate direction of electron flow. Solid lines designate experimentally assessed pathways, while dotted lines are proposed interactions. Red arrow is the previously-suggested Laz-AniA interaction, which is not supported by the data presented here. Red X indicates that, based on our data, Laz is not a surface-displayed protein. CcoN/O/P is cytochrome C oxidase *cbb*_3_. Red-tinted proteins contain heme redox centers, while blue colored proteins contain copper redox centers. CcoN, which contains both heme and copper redox centers, has a blue-to-red gradient. CCP, cytochrome *c* peroxidase. Figure adapted from Nobrega et al. (2016).

*N. gonorrhoeae*, the etiological agent of the sexually transmitted infection gonorrhea, causes significant morbidity globally (Newman et al., 2015). With the loss of ceftriaxone as the only remaining reliable monotherapy against gonorrhea (Unemo and Shafer, 2014; Unemo et al., 2016a; Lefebvre et al., 2018), development of new therapeutic interventions and gonorrhea vaccine(s) is vital. We recently proposed targeting anaerobic respiration to generate new antibiotics and demonstrated that nitrite reductase AniA activity could be abrogated by peptide inhibitors that also prevented *N. gonorrhoeae* from growing anaerobically (Sikora et al., 2017). AniA is considered a gonorrhea vaccine candidate (reviewed in Rice et al., 2017). It is a surface-displayed copper-containing lipoprotein which is essential for gonococcal viability during anaerobiosis (Mellies et al., 1997; Householder et al., 1999; Sikora et al., 2017). It is also important in biofilm formation and enhances bacterial survival during exposure to human serum (Cardinale and Clark, 2000; Ku et al., 2009; Shewell et al., 2013, 2017; Zielke et al., 2016; Sikora et al., 2017). Importantly, animals immunized with purified AniA protein generate functional antisera that are capable of blocking the nitrite reductase function in a whole cell assay and cross-react with a wide-range of gonococcal isolates (Shewell et al., 2013; Sikora et al., 2017). Laz donates electrons to gonococcal cytochrome *c* peroxidase (Ccp) and has been proposed, but not experimentally tested, to act as an electron donor to AniA (Boulanger and Murphy, 2002; Nobrega et al., 2016; Figure 1). In quantitative, high-throughput proteomics, we detected Laz in the cell envelopes and outer membrane vesicles (OMVs) isolated from four different gonococcal isolates and observed that Laz expression increases ∼3-fold during anaerobiosis (Zielke et al., 2014, 2016). The surface display of Laz in *N. meningitidis* and the estimated 31% effectiveness of an OMV meningococcal group B vaccine against gonorrhea (Deeudom et al., 2015; Petousis-Harris et al., 2017) prompted us to evaluate Laz as a gonorrhea vaccine antigen. In addition, we characterized the role of Laz within the gonococcal cell envelope and experimentally challenged the proposed Laz-AniA interaction. Finally, we optimized and performed chemical cross-linking followed by immunoblotting to covalently link interacting protein partners in living gonococci. We validated this experimental approach – applied for the first time in *N. gonorrhoeae* research – to interrogate protein complexes and demonstrate the appearance of the trimer form of AniA, as well as the formation of the β-barrel assembly machinery complex, in the presence of cross-linker.

## Materials and Methods

### Bacterial Strains and Growth Conditions

*Neisseria gonorrhoeae* FA1090 (Cohen et al., 1994) was used as wild type (WT). Replacement of the *laz* gene (*ngo0994*) with a kanamycin resistance cassette, and generation of an inducible complemented strain with *laz* expression under the control of the P_lac_ promoter, was performed previously (Zielke et al., 2016). A panel of clinical isolates collected from two public health clinics in Baltimore from 1991 to 1994 (LGB1, LG14, LG20, LG26) (Zielke et al., 2016); the Public Health-Seattle & King County Sexually Transmitted Disease clinic from 2011 to 2013 (UW01-UW13) (Zielke et al., 2016); and the 2016 WHO reference strains (Unemo et al., 2015, 2016b; Zielke et al., 2016) was used to examine expression of Laz. Glycerol stocks of strains frozen at -80°C were streaked onto gonococcal base agar (GCB, Difco) supplemented with Kellogg’s supplement I and 12.5 μM ferric nitrate (Spence et al., 2008) and incubated in a 5% CO_2_ atmosphere at 37°C for 18–20 h. Transparent, non-piliated colonies were subcultured onto GCB. Except where otherwise indicated, colonies were collected from plates with a polyester-tipped sterile applicator (Puritan) after 18–20 h incubation and suspended to an OD_600_ of 0.1 in gonococcal base liquid medium (GCBL) supplemented with 0.042% sodium bicarbonate and Kellogg’s supplements as above (Kellogg et al., 1963; Spence et al., 2008). Liquid cultures were propagated at 37°C with shaking (220 rpm) for 3 h, back-diluted to an OD_600_ of 0.1 in supplemented GCBL, and cultured in the same manner.

### Expression of Laz and AniA During Murine Infection

Five BALB/C mice were infected vaginally with strain FA1090 as described (Song et al., 2008) and vaginal washes were collected on days 1, 3, and 5 post-bacterial inoculation by pipetting 40 μL of sterile PBS in and out of the vaginas. This procedure was repeated three times for each mouse, and the resultant fluids from each time point were pooled.

### SDS-PAGE and Immunoblotting

Samples were normalized by OD_600_ values (whole cell lysates), protein concentration (subcellular fractionation samples), or colony forming units (CFU/mL; murine vaginal washes). Proteins were separated by sodium dodecyl sulfate – polyacrylamide gel electrophoresis (SDS-PAGE) on 4–12% Novex NuPAGE (Thermo Fisher Scientific), 4–15% Bio-Rad Criterion TGX (Bio-Rad), or Bio-Rad AnykD Criterion TGX (Bio-Rad) gels and visualized by colloidal Coomassie G-250 staining. Immunoblots were performed as described (Zielke et al., 2016; Sikora et al., 2017, 2018; Wierzbicki et al., 2017). The polyclonal rabbit anti-Laz antiserum used in the current study was generated previously by immunization with recombinant His-tagged Laz (Zielke et al., 2016).

### Exposure to Host-Relevant Conditions

FA1090 (WT), Δ*laz*, and Δ*laz*/P_lac_::*laz* cultured in GCBL were diluted to an OD_600_ of 0.2 and serially diluted in GCBL. Spots (5 μL) from each dilution were plated on GCB either supplemented as above or with the addition of 7.5% normal human serum (NHS) or 5 μM of the iron chelator deferoxamine mesylate (desferal), but no ferric nitrate. Plates were maintained for 22 h in 5% CO_2_, or for 48 h at 37°C in an anaerobic jar with BD GasPaks (BD) in the presence of 1.2 mM sodium nitrate as an electron acceptor. CFU/mL counts were enumerated after incubation as indicated.

### Sensitivity to Reactive Nitrogen Species

Rapidly growing cultures of strains indicated were standardized to an OD_600_ of 0.2, serially diluted, and spotted onto GCB supplemented with 0.1 mM IPTG and either sodium nitrite (0, 1.2, 2, 4, 8, or 16 mM) or the nitric oxide (NO) generator sodium nitroprusside (SNP; 0, 0.05, 0.1, 0.2, 0.4, 0.8, or 1.6 mM). CFUs were counted after 20–22 h incubation at 37°C in a 5% CO_2_ atmosphere. Experiments were performed on three separate occasions and percent survival relative to the reactive nitrogen species (RNS)-free condition is presented. Microscopy was performed with a Zeiss AxioObserver.D1 inverted microscope at 10× magnification 0.25 Phase Contrast 1 and at 2.5× magnification 0.06 phase contrast 1.

### Serum Sensitivity

Bacterial sensitivity to NHS was evaluated as described (Garvin et al., 2008). Strains were cultured in GCBL for 3 h at 37°C, diluted to an OD_600_ of 0.05 in sterile PBS, and diluted 1:1000 in Eagle’s minimal essential medium (EMEM). Seventy microliters of bacterial suspension (∼10^3^ CFU) were combined with 70 μL of EMEM, NHS, or heat-inactivated NHS (inactivated at 56°C for 30 min; final serum concentrations, 0 and 50%) in a 96 well microtiter plate. Microtiter plates were incubated at 37°C in a 5% CO_2_ environment for 1 h. Twenty microliters from each well were subsequently spotted onto GCB for CFU/mL enumeration.

### Etest Antimicrobial Sensitivity Testing

Bacterial susceptibility to 10 antimicrobials was assessed with Etests (bioMérieux) as directed by the manufacturer using non-piliated colonies of WT, Δ*laz*, and Δ*laz*/P_lac_::*laz* bacteria. Test strips were placed on plate surfaces and minimal inhibitory concentrations (MICs) were determined after ∼22 h incubation. Each experiment was performed on three separate occasions using fresh bacterial cultures and the consensus MICs obtained in at least two trials are reported.

### Subcellular Fractionation

Fractions corresponding to the cytoplasm/periplasm (C/P), cell envelope (CE), naturally secreted OMVs, and soluble supernatant (SS) were collected as described previously (Zielke et al., 2014, 2016) from 500 mL mid-logarithmic cultures. Supernatants were separated from bacteria by centrifugation, filtered through a 0.2 μm filter (VWR), and treated with DNaseI (New England Biolabs) and ethylenediaminetetraacetic acid-free Pierce protease inhibitor tablets (Thermo Fisher Scientific). Ultracentrifugation was used to isolate native OMVs. Soluble proteins were precipitated from OMV-free supernatants by treatment with 15% trichloroacetic acid and washed with ice-cold acetone. CE fractions were isolated by cold sodium carbonate extraction and ultracentrifugation of cell lysates.

### Assessments of Surface Exposure

Laz surface exposure was evaluated by spotting intact or lysed cells on nitrocellulose and immunoblotting as previously described (Zielke et al., 2016). Surface exposure was also examined by proteolytic shaving of intact gonococci as described (Zielke et al., 2016). Briefly, intact cells were suspended in PBS with 5 mM MgCl_2_ and treated with 0, 20, or 40 μg/mL proteinase K for 1 h at 37°C. Digestion was halted by the addition of 10 μL of 50 mM phenylmethanesulfonylfluoride and cells were washed with PBS. Cells were lysed in sample buffer, separated by SDS-PAGE and either probed with anti-Laz, anti-BamD, or anti-Zwf antiserum or visualized by Coomassie brilliant blue G250 staining to assess digestion and to ensure cells were not lysed during protease treatment. To evaluate digestion of periplasmic proteins, proteolysis was carried out in the same manner, with the exception that the outer membrane was disrupted by an extensive PBS wash prior to suspension in PBS-MgCl_2_.

### LOS Isolation and Silver Staining

Lipooligosaccharide (LOS) was extracted from liquid cultures standardized to an OD_600_ of 0.2 (Hitchcock and Brown, 1983; Wierzbicki et al., 2017). Cells were lysed by boiling in Laemmli buffer, cooled to room temperature, and proteins were digested by proteinase K treatment at 60°C for 1 h. LOS was subjected to SDS-PAGE with an 18% gel and silver stained (Tsai and Frasch, 1982).

### Biofilm Characterization

The ability of WT and Δ*laz* bacteria to form biofilms was examined as described (Anderson et al., 2016), with the exception that bacterial suspensions were prepared in GCBL. A BioTek Synergy HT plate reader (BioTek) was used to measure biofilm mass at 550 nm.

### *In vivo* Cross-Linking

To evaluate interactions between Laz and AniA, an *in vivo* cross-linking protocol (Gray et al., 2011) was adapted for *N. gonorrhoeae*. Non-piliated bacteria were collected from GCB and suspended to a final OD_600_ of 1.5 in 4 mL sterile PBS. Suspensions were centrifuged, supernatants were decanted, and pellets were suspended in 500 μL sterile PBS. Dithiobis(succinimidyl propionate) (DSP; Sigma) was hydrated to 25 mM in dimethylsulfoxide and added to 100 μL of each suspension to 0, 0.1, 0.25, or 0.4 mM. Cross-linking reactions were incubated at 37°C and 5% CO_2_ for 30 min and quenched by the addition of 50 mM Tris-HCl pH 8.0 and incubation at room temperature for 10 min. Cells were lysed by boiling in sample buffer without reducing agent to avoid cleaving DSP-linked complexes. SDS-PAGE separation was performed at 4°C and 150 V followed by immunoblotting.

### Bioinformatic Analyses

The amino acid sequence of Laz was used to query the NCBI non-redundant protein database to search for homologs. Amino acid sequences were aligned with the ClustalOmega online tool to generate a distance matrix and with ClustalW in MEGA7 software to examine phylogenetic relationships between homologs. A maximum likelihood tree was constructed in MEGA, using the Jones-Taylor-Thornton model to generate a pairwise distance matrix (Jones et al., 1992). Neighbor-Join and BioNJ algorithms were applied to the matrix to generate the initial tree, which was then heuristically searched with the Nearest-Neighbor-Interchange method. Phylogenies were tested with 500 bootstrap replications. Single nucleotide polymorphism analysis was performed by querying the *Neisseria* Multilocus Sequence Typing Database (PubMLST^1^) with the nucleotide sequence of the *laz* gene (NEIS1462, allele 15) against the 45,169 isolates in the database as of March 24, 2018. Phylogenetic analysis of the alleles across all *Neisseria*, as well as within* N. gonorrhoeae*, was performed as above.

### Statistical Analyses

GraphPad Prism software (version 6.0h for Mac OS X) was used for all statistical analyses. Built-in *t*-test analyses were used to test for statistical significance at *p* < 0.05. For bacterial sensitivity to RNS, the built-in “Multiple *t*-tests – one per row” analysis was performed, and statistical significance was determined using the Holm–Sidak method to correct for multiple comparisons. Each row (nitrite/nitric oxide concentration) was analyzed individually. Alpha was set to 0.05.

### Ethics Statement

Animal experiments were conducted at the Uniformed Services University of the Health Sciences (USUHS) according to the guidelines of the Association for the Assessment and Accreditation of Laboratory Animal Care under protocol # MIC16-488 that was approved by the University’s Institutional Animal Care and Use Committee. The USUHS animal facilities meet the housing service and surgical standards set forth in the “Guide for the Care and Use of Laboratory Animals” NIH Publication No. 85-23, and the USU Instruction No. 3203, “Use and Care of Laboratory Animals.” Animals are maintained under the supervision of a full-time veterinarian.

## Results and Discussion

### Laz Conservation

**FIGURE 2:**
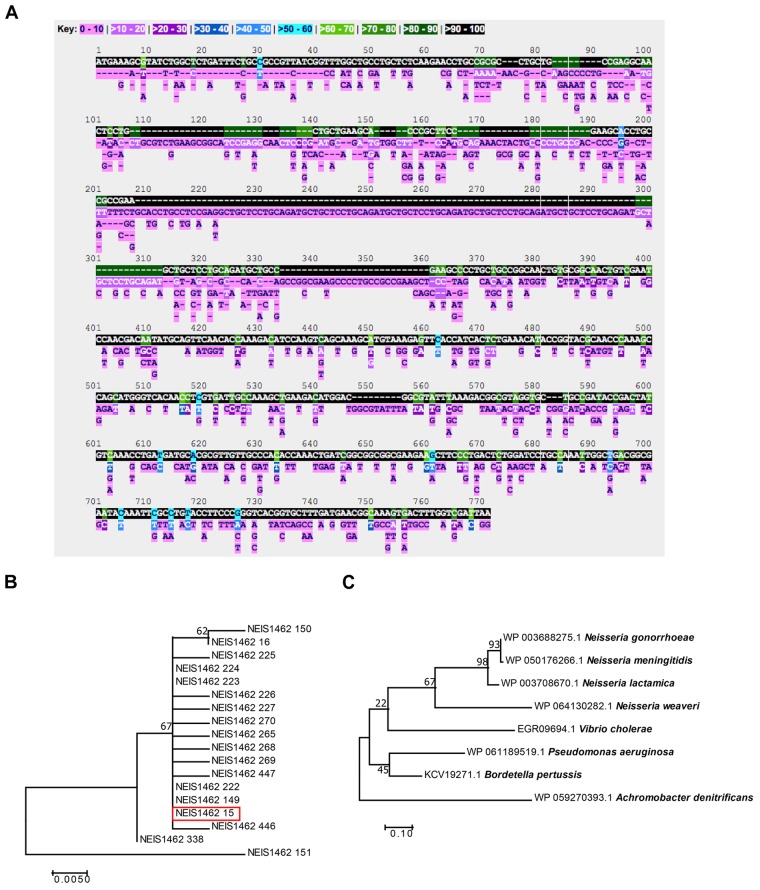
Bioinformatic analysis of Laz conservation. **(A)** The PubMLST *Neisseria* database was queried for nucleotide polymorphisms and alleles of the *laz* (NEIS1462) locus among the 45,169 *Neisseria* isolates in the database. **(B)** A phylogenetic tree was constructed in MEGA using translated nucleic acid sequences of all *laz* alleles among *N. gonorrhoeae* in the PubMLST *Neisseria* database. **(C)** A phylogenetic tree was constructed in MEGA with amino acid sequences downloaded from NCBI for Laz/Azurin homologs. For both phylogenies, Neighbor-Join and BioNJ algorithms generated the initial phylogenetic tree for heuristic searches. To test the phylogenies, 500 bootstrap replicates were performed. Highest log-likelihood trees are presented.

To evaluate Laz as a potential gonorrhea vaccine candidate, we first examined the level of conservation between Laz homologs within all *Neisseria* species with sequences available on the PubMLST *Neisseria* database. Five-hundred three alleles, with 602 polymorphic sites, were discovered among the 45,169 sequences in the database. Stretches of identical sequence were found in 90–100% of the alleles; however, several portions were conserved in only 70–80 or 80–90% of alleles (Figure 2A).

To analyze Laz diversity among *N. gonorrhoeae* and other *Neisseria* species further, phylogenetic trees were constructed using the translated amino acid sequences from all 18 alleles found in *N. gonorrhoeae* (Figure 2B), as well as the 503 alleles found in all available *Neisseria* isolates (Supplementary Figure S1). This analysis indicated that Laz alleles within *N. gonorrhoeae* are closely related, with the exception of allele 151, which formed an outgroup. Allele 15, of which FA1090 Laz is a member (Figure 2B, red box), represented the majority of alleles found in *N. gonorrhoeae* (3,543 out of 4,924 *N. gonorrhoeae* isolates; 1,047 isolates have no value for the *laz* locus).

To assess the cross-species conservation of Laz, we compared the amino acid similarity between Laz homologs in some Gram-negative bacteria and other members of the *Neisseria* genus. In agreement with previous studies (Sutherland and Wilkinson, 1963), Laz homologs could not be identified in *E. coli, Klebsiella pneumoniae, Haemophilus influenzae, H. ducreyi, Shigella flexneri, Chlamydia trachomatis*, or *Treponema pallidum*. Laz homologs were relatively well-conserved, with amino acid identities greater than 37% in all homologs identified (Table 1). Homologs were further scrutinized for the presence of a lipoprotein signal peptide using the LipoP 1.0 server. If no signal peptide was detected, the SignalP 4.1 server was used to search for a sequence recognized by signal peptidase I. In a phylogenetic analysis of Laz homologs, *Vibrio cholerae* azurin formed a cluster with the *Neisseria* species apart from *P. aeruginosa* and *Bordetella pertussis*, while *Achromobacter denitrificans* formed an outgroup (Figure 2C).

**Table 1:** Amino acid identity of Laz homologs.

Organism with accession no.	Amino acid identity	Predicted lipoprotein signal peptide?	Molecular mass (kDa)^b^
*Neisseria gonorrhoeae* FA1090 [WP_003688275.1]	100%	Yes	18.5
*Neisseria meningitidis* [WP_050176266.1]	99.45%	Yes	18.5
*Neisseria lactamica* [WP_003708670.1]	87.98%	Yes	18.9
*Neisseria weaveri* [WP_064130282.1]	53.89%	Yes	19.2
*Achromobacter denitrificans* [WP_059270393.1]	37.32%	No	15.9
*Vibrio cholerae* [EGR09694.1]	46.96%	No^a^	12.5
*Pseudomonas aeruginosa* [WP_061189519.1]	51.08%	No	16.0
*Bordetella pertussis* [KCV19271.1]	52.17%	No	15.1


*^*a*^No signal peptide detected. ^*b*^Mass calculated from unprocessed primary amino acid sequence.*

These comprehensive analyses showed the high degree of conservation for Laz, with a single allele representing greater than 90% of *N. gonorrhoeae* Laz sequences in the PubMLST *Neisseria* database, and demonstrated that azurin proteins are closely related in divergent bacteria.

### Expression of Laz Among Gonococcal Clinical Isolates and Within *Neisseria*

**FIGURE 3:**
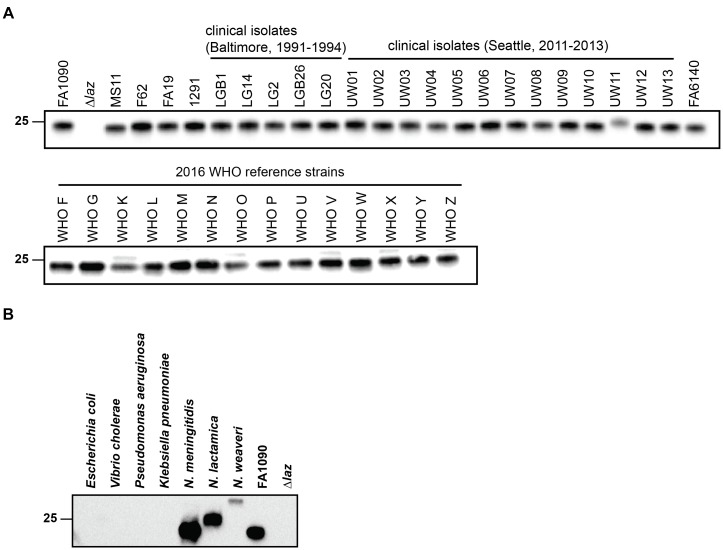
Anti-Laz antiserum recognizes Laz in genetically, geographically, and temporally distinct clinical *N. gonorrhoeae* isolates and in other *Neisseria* species. **(A)** Common laboratory strains; clinical isolates from Baltimore between 1991 and 1994 and from Seattle between 2011 and 2013; and the 2016 WHO reference strains were cultured on solid medium for 20 h in a 5% CO_2_ environment at 37°C. Whole cell lysates from all strains were collected and subjected to immunoblotting analysis with anti-Laz antiserum. **(B)** Immunoblotting analysis of whole cell lysates of *E. coli* BL21 (DE3); *Vibrio cholerae* N19691; *Pseudomonas aeruginosa* PA01; *Klebsiella pneumoniae* 6069; *N. meningitidis* MC58; the commensal bacterium *Neisseria lactamica* NLI83/-01; and the opportunistic pathogen *Neisseria weaveri* 1032. Lysates were standardized by OD_600_ values, separated by SDS-PAGE on a 4–15% Tris-glycine gel, and probed with anti-Laz polyclonal rabbit antiserum. FA1090 and Δ*laz* were included as positive controls. OD_600_, optical density at 600 nm; SDS-PAGE, sodium dodecyl sulfate-polyacrylamide gel electrophoresis. Migration of the closest molecular mass marker is indicated in kDa for each blot.

Laz was similarly expressed in the CE of four laboratory gonococcal strains examined by proteomics (Zielke et al., 2014). We assessed Laz cellular pools across a panel of 38 clinical isolates, collected at different locations and during different time periods. Immunoblotting analysis indicated that Laz was expressed at similar levels in all isolates, with the exception of UW11, in which expression was slightly lower and the protein migrated at a higher molecular weight, suggesting the presence of additional post-translational modification(s). We considered that the faint band observed above Laz in WHO strains K, O, V, X, and Z may represent a small portion of Laz that has not undergone the full lipoprotein maturation pathway, a subset of post-translationally modified Laz, or an unidentified cross-reactive protein specific to those five strains (Figure 3A).

We next probed whole cell lysates of seven different Gram-negative bacteria with antiserum against recombinant, His-tagged gonococcal Laz lacking the signal peptide (Zielke et al., 2016) to determine whether the antiserum would recognize homologous proteins in *Neisseria*, in addition to species from other genera. As predicted by our bioinformatic searches, Laz homologs were not recognized in *E. coli* or *K. pneumoniae*. Cross-reactive bands were detected only in the *Neisseria* species examined; neither *P. aeruginosa* nor *V. cholerae* azurin could be distinguished despite high (∼50%) amino acid identity (Figure 3B). *Neisseria weaveri* Laz migrated at a substantially higher apparent molecular weight than gonococcal Laz, despite predicted molecular weights within 0.7 kDa, suggesting that Laz may be post-translationally decorated in *N. weaveri*.

These studies further confirmed conservation and showed broad expression of Laz in pathogenic and commensal *Neisseria*.

### Subcellular Localization and Surface Accessibility of Laz

**FIGURE 4:**
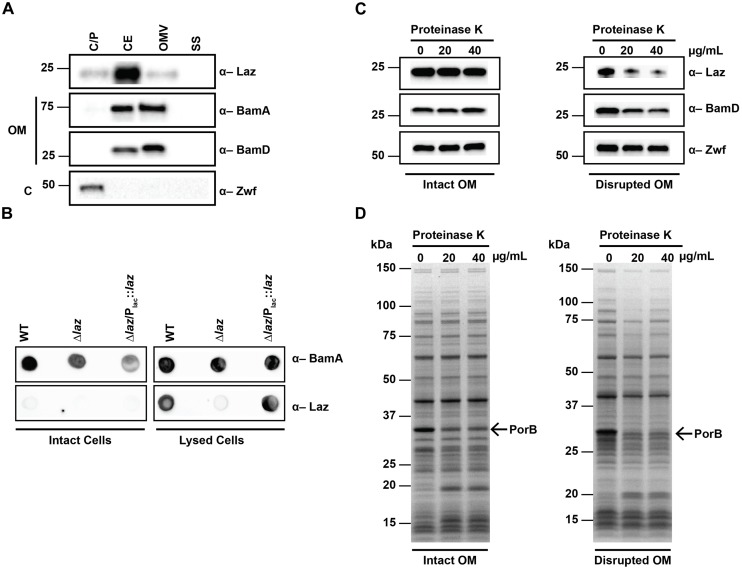
Subcellular localization of Laz. **(A)** Subcellular fractions containing cytoplasmic/periplasmic (C/P), cell envelope (CE), outer membrane vesicle (OMV), and soluble supernatant (SS) proteins of WT FA1090 were prepared by differential ultracentrifugation and sodium carbonate extraction, normalized based on protein concentration, separated by SDS-PAGE, and probed with indicated antisera. **(B)** Same amounts of intact (left panels) or lysed (right panels) cells of WT, isogenic knockout Δ*laz*, and Δ*laz*/P_lac_::*laz* were spotted onto nitrocellulose and incubated with indicated antisera. **(C,D)** Whole cells of WT FA1090 were subjected to proteolytic shaving with indicated concentrations of proteinase K. Outer membranes were disrupted by washing cells with PBS prior to proteolysis (right panels). Subsequent to proteinase treatment, cells were lysed in protein sample buffer, normalized by OD_600_ units, separated by SDS-PAGE, and either probed with anti-Laz, anti-BamD, or anti-Zwf antiserum **(C)**, or stained with Coomassie brilliant blue G250 **(D)**. OM, outer membrane; C, cytoplasm; SDS-PAGE, sodium dodecyl sulfate – polyacrylamide gel electrophoresis; OD_600_, optical density at 600 nm. Migration of the closest molecular mass marker is indicated in kDa for each blot.

To gain insights into the cellular location of Laz, we performed immunoblot analysis of cytoplasmic/periplasmic (C/P), cell envelope (CE), OMV, and soluble supernatant (SS) subcellular fractions, using control antisera against the surface-exposed integral outer membrane component of the β-barrel assembly machinery (BAM) complex, BamA (Ricci and Silhavy, 2012; Zielke et al., 2016); the periplasmic-facing outer membrane lipoprotein of the BAM complex, BamD (Misra, 2012; Sikora et al., 2018); and the cytoplasmic enzyme Zwf (Lundberg et al., 1999; Sandoval et al., 2011; Wierzbicki et al., 2017). This examination revealed that Laz predominantly localized to the CE, but could also be found in the C/P and OMV fractions. BamA was mostly found in the CE and OMV fractions with a small amount detected in the C/P, reflecting the presence of BamA polypeptide transport-associated domains residing in the periplasm (Sikora et al., 2018). BamD was exclusively detected in the CE and OMV fractions, while Zwf localized solely to the C/P fraction (Wierzbicki et al., 2017). None of the proteins examined were detected in the SS (Figure 4A).

Studies in *N. meningitidis* suggested that at least a subset of the cellular pool of Laz is exposed to the extracellular milieu (Deeudom et al., 2015). To determine whether Laz was surface-displayed in *N. gonorrhoeae*, we spotted intact and lysed cells onto nitrocellulose and probed the membranes with antisera against Laz and the surface exposed BamA (Zielke et al., 2016). While BamA was detected in both intact and lysed cells, Laz was observed only when cells were lysed (Figure 4B), suggesting that this protein is not surface exposed in *N. gonorrhoeae*. To further confirm this observation, we performed proteolytic shaving of intact cells with proteinase K (Zielke et al., 2016). Coomassie staining revealed digestion of surface proteins with increasing concentrations of protease, most notably in the predominant PorB band at approximately 35 kDa (Figure 4D, left panel). However, immunoblotting with anti-Laz antiserum indicated that Laz was not accessible to extracellular protease, similar to the periplasmic BamD and the cytoplasmic Zwf (Figure 4C, left panel). To verify Laz is facing the periplasm, we performed a similar experiment in which the outer membrane was disrupted by an additional PBS wash prior to proteolytic cleavage. Immunoblotting analysis revealed digestion of Laz and BamD, but not Zwf (Figure 4C, right panel). In combination with Coomassie staining (Figure 4D, right panel), our results showed that solely the outer membrane integrity in these cells was disrupted, which allowed the protease to access periplasmic targets but not cytoplasmic proteins.

The presence of Laz in the C/P fraction, in combination with the inability of anti-Laz antiserum to recognize Laz on intact cells and its resistance to externally added proteases – but not in cells in which the outer membrane integrity was compromised – provide compelling evidence that this protein faces the periplasmic space of the outer membrane in *N. gonorrhoeae*. This discovery eliminates Laz as a gonorrhea vaccine candidate and further highlights the necessity to verify homologous protein localization even between closely related species.

### Loss of Laz Increases Ampicillin Susceptibility

To determine whether deletion of Laz perturbed cell envelope homeostasis, we examined bacterial sensitivity to 10 antibiotics using Etest antimicrobial test strips. The Δ*laz* mutant exhibited a twofold decrease in MIC to the penicillin derivative ampicillin, which targets the bacterial cell wall (Table 2). Ampicillin resistance was restored to the WT level in the Δ*laz*/P_lac_::*laz* complementation strain. However, the Δ*laz* mutant was not more sensitive to vancomycin, which is typically too large to penetrate the Gram-negative outer membrane (Kohanski et al., 2010). Further, the antibiotics azithromycin and tetracycline, which must enter the cell to reach their ribosome targets (Kohanski et al., 2010), were not more effective against the Δ*laz* mutant. Bacteria lacking Laz were also not more susceptible to the cephalosporins cefotaxime or ceftazidime. Aminoglycosides are more effective against bacteria with disrupted membrane integrity, as illustrated by the synergy between aminoglycosides and β-lactam antimicrobials (Kohanski et al., 2010). However, the Δ*laz* mutant was not more sensitive to either gentamicin or tobramycin.

**Table 2:** Etest assessment of WT, Δ*laz*, and Δ*laz*/P_lac_::*laz* MIC values.

	FA1090 (WT)^a^	Δ*laz*^a^	Δ*laz*/P_lac_::*laz*^a^
Polymyxin B	64	64	64
Azithromycin	0.064	0.064	0.5^b^
Cefotaxime	0.004	0.004	0.004
Ampicillin	0.125	0.064	0.125
Tetracycline	0.125	0.125	0.125
Benzylpenicillin	0.064	0.064	0.064
Vancomycin	8	8	8
Gentamicin	4	4	4
Tobramycin	8	8	8
Ceftazidime	0.032	0.032	0.032


*^*a*^MIC values are presented in *μ*g/mL. ^*b*^Construct used for complementation encodes erythromycin resistance, which also confers resistance to azithromycin.*

These results indicate that interference with electron transport due to loss of Laz results in a slight disruption of cell envelope homeostasis.

### Bacterial Viability During *in vitro* Growth Is Not Affected in the Δ*laz* Mutant

**FIGURE 5:**
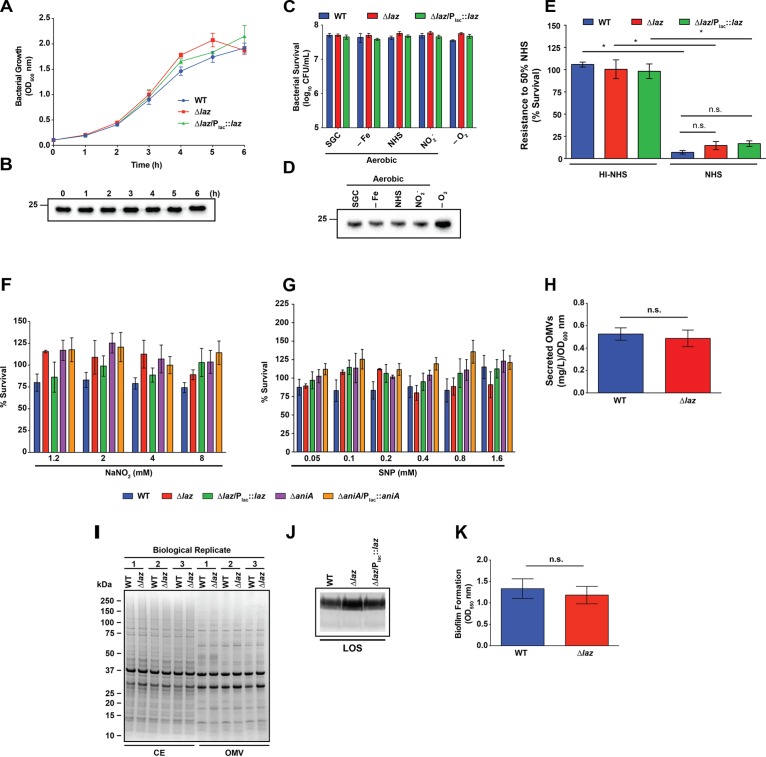
Phenotypic assessment of Laz. **(A)** WT, mutant, and complemented strains as indicated were cultured aerobically in liquid medium. Laz expression in the complemented strain was induced by addition of IPTG. Each h, bacterial growth was assessed by OD_600_ measurement. **(B)** Whole cell lysates, collected from liquid cultures at timepoints indicated, were separated by SDS-PAGE and probed with anti-Laz antiserum. **(C)** WT, isogenic knockout Δ*laz*, and Δ*laz*/P_lac_::*laz* were cultured in liquid medium under standard aerobic conditions for 3 h until OD_600_ had at least doubled. Cultures were standardized to an OD_600_ of 0.2, serially diluted, and spotted onto GCB prepared as normal (SGC) or supplemented with 5 μM desferal (–Fe), 7.5% normal human serum (NHS), or 1.2 mM NaNO_2_. All plates were cultured at 37°C in a 5% CO_2_ atmosphere for ∼20 h. A subset of nitrite-containing plates was maintained anaerobically at 37°C for 48 h (–O_2_). CFU were enumerated after appropriate incubation periods. **(D)** Whole cell lysates collected from plates cultured as in **(C)** were separated by SDS-PAGE and probed with anti-Laz antiserum. **(E)** Rapidly-growing cultures of WT, Δ*laz*, and Δ*laz*/P_lac_::*laz* were suspended to an OD_600_ of 0.05 in sterile PBS, diluted 1000-fold in EMEM, and combined with equal volumes of either normal- or heat-inactivated human serum. Bacteria were exposed to serum for 1 h at 37°C in a 5% CO_2_ atmosphere, after which each condition was spotted onto a GCB plate. CFU enumeration was performed after 20 h incubation at 37°C at 5% CO_2_. Results are presented as percent survival compared to bacteria treated in the same manner and incubated in EMEM instead of serum (*n* = 4, ±SEM). **(F)** Rapidly growing cultures of strains indicated were normalized to an OD_600_ of 0.2, serially diluted, and spotted onto GCB supplemented with 0.1 mM IPTG and with or without the indicated concentrations of NaNO_2_. CFUs were enumerated after 20–22 h incubation, and the percent survival was calculated compared to the 0 mM NaNO_2_ condition. **(G)** Strains were prepared as in **(F)** and spotted onto GCB supplemented with 0.1 mM IPTG and with or without the indicated concentrations of SNP. Percent survival was calculated compared to the 0 mM SNP condition after 20–22 h incubation. **(H)** OMVs from the supernatants of WT and Δ*laz* liquid cultures were collected by ultracentrifugation and quantified by protein concentration. **(I)** CE and OMV fractions collected from WT and Δ*laz* were standardized by protein concentration and separated by SDS-PAGE. Proteins were visualized by Coomassie brilliant blue staining. Isolation was performed three times, and all three replicates are shown. Migration of molecular mass markers is shown on the left in kDa. **(J)** LOS was isolated from rapidly growing liquid cultures of WT, isogenic knockout Δ*laz*, and complementation strain Δ*laz*/P_lac_::*laz*. Cells were lysed by boiling in buffer containing SDS and proteins were digested by proteinase K treatment. LOS was separated by SDS-PAGE and silver stained. **(K)** WT and isogenic knockout Δ*laz* were suspended to an OD_550_ of 1.5 in supplemented GCBL and added to 96-well microtiter plates. Bacteria were cultured without shaking at 37C in a 5% CO_2_ atmosphere for 24 h. Planktonic bacteria and spent medium were removed and biofilms were washed once with PBS. Plates were dried at room temperature and stained by the addition of 0.1% crystal violet in 2% ethanol, then washed 3 times with PBS. Stained biofilms were dried overnight at room temperature, dissolved in 30% acetic acid, and quantified by OD_550_ measurement. Biofilm experiments were performed three times, each with 4 or 8 technical replicates, for a total of 20 datapoints. Mean ± SEM is presented. Unless otherwise specified, all experiments were performed on three separate occasions and mean ± SEM is presented; ^∗^*p* < 0.05. IPTG, isopropyl β-D-thiogalactopyranoside; OD_600_, optical density at 600 nm; OD_550_, optical density at 550 nm; SNP, sodium nitroprusside; GCB, gonococcal base medium; GCBL, gonococcal base liquid medium; PBS, phosphate buffered saline; EMEM, Eagle’s minimal essential medium; CE, cell envelope; OMV, outer membrane vesicle; SDS-PAGE, sodium dodecyl sulfate-polyacrylamide gel electrophoresis; CFU, colony forming unit; LOS, lipooligosaccharide. Migration of the closest molecular mass marker is indicated in kDa for each blot.

We next sought to assess the consequences of Laz deletion on gonococcal fitness during *in vitro* growth, and its expression characteristics. We saw no difference in growth kinetics during standard aerobic liquid culture between the WT, Δ*laz*, or Δ*laz*/P_lac_::*laz* strains (Figure 5A). Expression analysis indicated that the cellular pool of Laz was consistent across all stages of growth (Figure 5B). Growth of *N. gonorrhoeae* in liquid medium is not representative of all environmental stressors encountered in the host, including microaerobic or anaerobic conditions (Smith, 1975; Knapp and Clark, 1984; Edwards and Apicella, 2004), iron restriction (Ducey et al., 2005; Cornelissen, 2008; Noto and Cornelissen, 2008), or the presence of serum (Clark et al., 1988; Edwards and Apicella, 2004). The flow of electrons via the transport chain could be important during colonization of different microecological niches in the human host. However, we observed no difference in the viability of the Δ*laz* mutant compared to WT during growth under iron limitation (-Fe), exposure to 7.5% NHS, or anaerobiosis (-O_2_), suggesting that other electron donor(s) may compensate for Laz in the electron transport chain (Figures 1, 5C). In support of our proteomics investigation (Zielke et al., 2016), immunoblotting analysis revealed that Laz was upregulated during anoxia but not under other conditions relevant to infection (Figure 5D). Similarly, Laz is upregulated in response to microaerobic and microaerobic denitrifying conditions in *N. meningitidis* but is not essential for growth under anaerobic conditions (Woods et al., 1989; Deeudom et al., 2015).

Expression of AniA increases gonococcal resistance to NHS; therefore, as the first means of evaluating the hypothesized AniA-Laz interaction, we assessed bacterial sensitivity to NHS in liquid medium (Garvin et al., 2008). Exposure to 50% serum resulted in a significant decrease in viability for all strains when compared to bacteria exposed to the same concentration of heat-inactivated NHS (Figure 5E). No significant difference was observed between the viability of WT, Δ*laz*, or complemented bacteria during serum exposure (Figure 5E).

We also evaluated the sensitivity of Laz- or AniA-deficient bacteria to the RNS nitrite and NO. The loss of either protein did not significantly alter bacterial viability during exposure to sub-lethal concentrations of sodium nitrite (NaNO_2_; Figure 5F) or the NO generator SNP (Anjum et al., 2002; Figure 5G). Microscopic examination of bacterial spots on medium containing 16 mM nitrite revealed that all strains were killed after a short period of growth, which resulted in dilution spots with hazy, difficult-to-define borders at low suspension dilutions. Higher dilutions contained miniscule colonies that were nearly indistinguishable from insoluble starch in the medium (Supplementary Figure S2). For this reason, bacterial survival was not calculated at 16 mM nitrite, although the high nitrite level did not appear to disproportionately affect any of the strains. Both mutants tested possess an intact nitric oxide reductase (NorB). Gonococcal NorB rapidly establishes a steady-state NO level as a function of either nitrite or NO donor concentration and maintains NO concentrations associated with anti-inflammatory effects in the host (<1 μM; Cardinale and Clark, 2005). Our results indicate that neither Laz nor AniA is critical for proper NorB function. We also demonstrate that *N. gonorrhoeae* is intrinsically more resistant to nitrosative stress than *N. meningitidis*. A WT serogroup B meningococcal strain failed to grow during exposure to 1.0 mM SNP (Anjum et al., 2002), while in our experiments, the viability of WT *N. gonorrhoeae* was unaffected by 1.6 mM of the NO generator. Our results likely reflect adaptations developed due to the different host niches the two species occupy.

### Laz Deletion Does Not Affect OMV Production or Cell Envelope Composition

The Δ*laz* mutant exhibits a low level of cell envelope stress, as demonstrated by its enhanced susceptibility to ampicillin (Table 2). Outer membrane stress has been hypothesized to contribute to increases in the release of OMVs (Kulp and Kuehn, 2010; Kulkarni and Jagannadham, 2014; Haurat et al., 2015). OMVs are utilized as vaccines against many pathogenic bacteria and their increased production is of interest from a manufacturing standpoint (van der Pol et al., 2015; Jan, 2017). We therefore examined whether the stress induced by removal of Laz would enhance OMV formation. Loss of Laz was not associated with a significant difference in OMV production during standard liquid culture (0.53 ± 0.06 mg L^-1^OD_600_^-1^ for WT compared to 0.49 ± 0.07 mg L^-1^OD_600_^-1^ for Δ*laz*; Figure 5H). Further, the CE and OMV protein profiles were not noticeably altered in the Δ*laz* mutant, as assessed by Coomassie staining (Figure 5I).

To determine whether the Δ*laz* mutant’s increased susceptibility to ampicillin was due to alterations in LOS structure, we isolated crude LOS from WT, Δ*laz*, and Δ*laz*/P_lac_::*laz* bacteria (Hitchcock and Brown, 1983; Wierzbicki et al., 2017). However, no differences in the overall LOS structure were apparent (Figure 5J).

Cumulatively, our observations that the Δ*laz* mutant was not more sensitive to most antibiotics tested, WT and Δ*laz* bacteria produced similar amounts of OMVs, no gross changes in CE or OMV protein composition were associated with Laz deletion, and LOS structure was not altered in the *laz* null mutant indicated that the absence of Laz was minimally disruptive to cell envelope integrity.

### Biofilm Formation Is Not Altered in the Absence of Laz

*Neisseria gonorrhoeae* forms biofilms on primary urethral and cervical cells *in vitro* and *in vivo* (Greiner et al., 2005; Steichen et al., 2008) and members of the anaerobic regulon are upregulated in biofilms compared to planktonic bacteria (Falsetta et al., 2009; Phillips et al., 2012). Ccp, for which Laz is an electron donor (Figure 1; Nobrega et al., 2016), and AniA were also upregulated during biofilm growth (Falsetta et al., 2009; Phillips et al., 2012). We therefore sought to determine whether biofilm formation was affected in the Δ*laz* mutant. This assay did not reveal any difference in biofilm mass produced by the Δ*laz* mutant compared to WT bacteria (Figure 5K). Our findings are consistent with the involvement of AniA and Ccp in early stages of biofilm formation reported by others (Falsetta et al., 2009), and suggest that Laz and the two other electron transport proteins are dispensable during early stages of gonococcal biofilm formation (Anderson et al., 2016).

### Laz and AniA Are Discordantly Expressed During Genital Tract Infection of Female Mice

**FIGURE 6:**
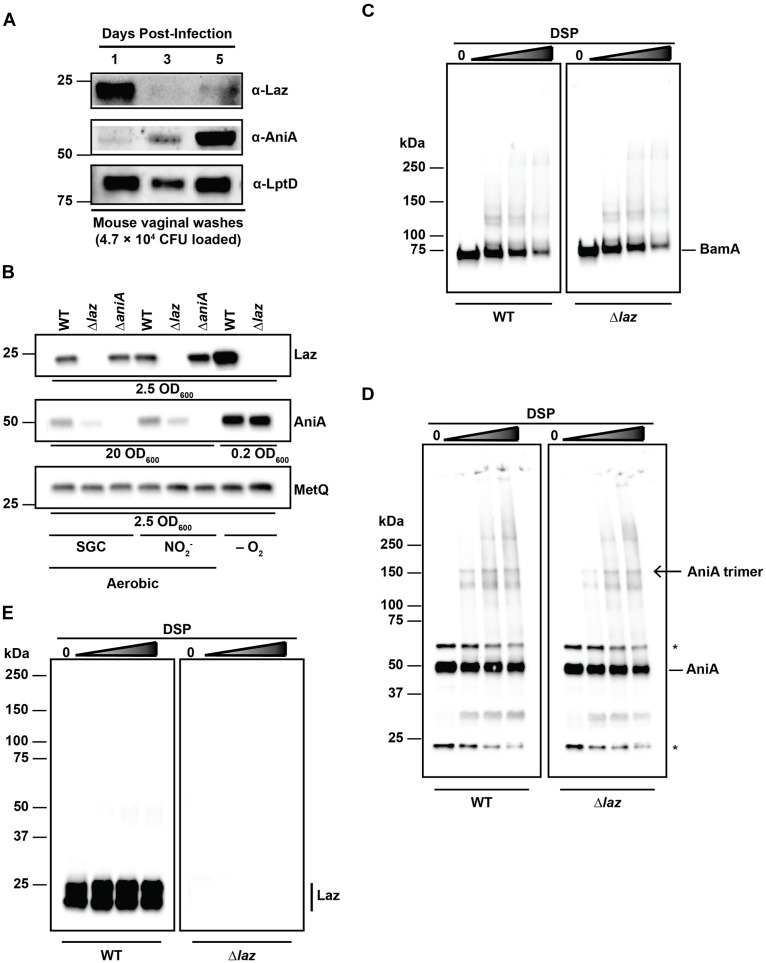
Interaction studies between Laz and AniA. **(A)** Vaginal wash samples collected and pooled from female mice infected with WT bacteria after 1, 3, and 5 days post-inoculation were standardized by total CFU count (4.7 × 10^4^ CFU), separated by SDS-PAGE, and probed with anti-Laz, anti-AniA, or anti-LptD antisera, as indicated. **(B)** Whole cell lysates of WT and isogenic knockouts Δ*laz* and Δ*aniA* were collected from solid medium after incubation aerobically under standard growth conditions (SGC) and either anaerobically (–O_2_) or aerobically with nitrite as a terminal electron acceptor (${\mathrm{NO}}_{2}^{-}$). Lysates were standardized by OD_600_ values, separated by SDS-PAGE, and probed with anti-Laz, anti-AniA, or anti-MetQ antisera as indicated. Experiment was performed three times and representative blots are presented. Migration of the closest molecular mass marker is indicated in kDa for each blot in **(A,B)**. **(C–E)** Intact WT or isogenic knockout Δ*laz* bacteria cultured under standard aerobic conditions were collected from GCB, washed twice with PBS, and incubated with increasing concentrations of DSP at 37°C and 5% CO_2_ for 30 min to cross-link interacting proteins. Cross-linked samples were standardized by OD_600_ value, separated by SDS-PAGE at 4°C in the absence of a reducing agent, and probed with anti-BamA **(C)**, anti-AniA **(D)**, or anti-Laz (**E**; indicated by a vertical bar) antisera. **(D)** Non-specific cross-reactive bands are noted with an asterisk (^∗^). The homotrimer form of AniA is noted with an arrow. **(E)** Lower molecular weight band is also Laz, as indicated by the lack of signal in the Δ*laz* blot. This band is likely a folded version of the protein, as a disulfide bond between Cys82 and Cys59 is formed in the mature protein (PDB ID: 3AY2) and remains intact without reducing agent. Migration of molecular mass markers (in kDa) is noted on the left of each pair of immunublots, and the migration of each antigen is noted on the right. GCB, gonococcal base medium; SDS-PAGE, sodium dodecyl sulfate – polyacrylamide gel electrophoresis; OD_600_, optical density at 600 nm; DSP, dithiobis(succinimidyl propionate).

Laz and AniA are transcribed during infection of the female lower genital tract (McClure et al., 2015). Additionally, sera from women with gonococcal infections cross-react with AniA, indicating that this protein is expressed *in vivo* (Clark et al., 1988). However, post-translation protein levels of Laz have not been assessed. Therefore, to examine the expression of Laz *in vivo*, as well as to provide support for our strategy of targeting anaerobic respiration for the development of new therapeutics against gonorrhea (Sikora et al., 2017), we inoculated 17-β estradiol-treated BALB/c mice vaginally with WT bacteria and collected vaginal washes on days 1, 3, and 5 post-infection to assess the expression of Laz and AniA during colonization. In the same set of vaginal wash samples, from which 4.7 × 10^4^ CFUs were loaded, we found that Laz was highly expressed on the first day of infection, then at low levels at days 3 and 5, while the abundance of AniA increased over the course of the infection and was highly expressed on day 5 (Figure 6A). In contrast, an essential outer membrane protein and gonorrhea vaccine antigen LptD (Zielke et al., 2014, 2016; El-Rami et al., 2018), was detected at similar levels on days 1 and 5 and slightly lower at day 3 of infection. Quantitative cultures of gonococci isolated during murine infection have demonstrated that *N. gonorrhoeae* replicates over the course of the infection (Jerse, 1999). Additionally, for the experiment presented here, the number of bacteria increased from day 1 (2.87 × 10^6^ CFU/mL) to day 3 (4.45 × 10^6^ CFU/mL), then slightly decreased on day 5 (2.08 × 10^6^ CFU/mL), consistent with previous reports that found reduced colonization in the middle of the infection due to hormonal factors (Jerse, 1999; Song et al., 2008; Cole et al., 2010). Our results demonstrated for the first time that Laz and the vaccine antigens AniA and LptD are expressed by replicating gonococci within the murine lower reproductive tract.

### Absence of Laz Influences AniA Expression

Our observation that Laz and AniA exhibited apparently opposing expression dynamics during infection of the female mouse prompted us to examine the expression of each protein in the absence of the other protein. Whole cell lysates derived from WT, Δ*laz*, and Δ*aniA* cultured under different conditions were probed with antisera against Laz, AniA, or MetQ as a control. AniA is essential for bacterial survival anaerobically (Mellies et al., 1997; Householder et al., 1999; Sikora et al., 2017), which precluded experiments with the Δ*aniA* mutant under anaerobic conditions. Immunoblotting analyses indicated that both AniA and Laz were upregulated during anoxia, as expected (Figure 6B). However, we found that the cellular pool of AniA decreased in the absence of Laz during aerobic growth, regardless of the presence of nitrite in the medium (Figure 6B).

### *In vivo* Cross-Linking Reveals No Interaction Between Laz and AniA

Laz has been proposed to act as an electron donor to AniA in *N. gonorrhoeae* (Boulanger and Murphy, 2002; Nobrega et al., 2016), although experiments in *N. meningitidis* and *P. aeruginosa* demonstrated that the respective nitrite reductases do not receive electrons from the azurin proteins (Vijgenboom et al., 1997; Aas et al., 2015). Our studies showed that Laz is a bona fide periplasmic lipoprotein (Figures 4B,C) whereas AniA is exposed to the extracellular milieu (Ku et al., 2009; Shewell et al., 2013, 2017; Zielke et al., 2016), which would hinder the Laz-AniA interaction. To challenge the hypothesis that Laz and AniA are interacting partners in the gonococcus, we decided to pursue the capture of protein–protein interactions in the cell wall of living cells by employing a crosslinking approach that has been used successfully in *E. coli* and *V. cholerae* but has not been reported for *N. gonorrhoeae* (Zgurskaya and Nikaido, 2000; Haft et al., 2007; Gray et al., 2011). First, we optimized DSP crosslinking by capturing protein–protein interactions within the five component BAM complex, which constitutes BamA, BamC, BamD, BamE, and RmpM in *N. gonorrhoeae* (Sikora et al., 2018). DSP contains two *N*-hydroxysuccinimide amine-reactive groups separated by a 12 Å spacer arm. The spacer contains a disulfide bond that can be cleaved in the presence of a reducing agent, allowing for reversible cross-linking. DSP is membrane permeable and is thus useful for interrogating intracellular protein–protein interactions. Primary amines, such as those found on the N-termini of proteins or on lysine side chains, will be covalently modified, capturing proteins that interact within 12 Å of each other (Zgurskaya and Nikaido, 2000; Haft et al., 2007; Gray et al., 2011). We treated living WT and Δ*laz* cells with increasing concentrations of DSP and monitored for the formation of protein complexes in the absence of a reducing agent (Figures 6C–E). As expected (Sikora et al., 2018), increased BAM complex formation was observed with higher concentrations of DSP in WT and Δ*laz* gonococci (Figure 6C). Subsequently, we examined potential AniA-Laz protein complexes using anti-AniA and anti-Laz antisera (Figures 6D,E). Numerous additional bands were apparent in the AniA blot in the presence of DSP and cross-linking readily increased in a dose-dependent manner (Figure 6D). AniA is a homotrimer in *N. gonorrhoeae* (Boulanger and Murphy, 2002; Sikora et al., 2017), and a band corresponding to the trimer form is apparent in the cross-linked samples (Figure 6D, open arrow). In contrast, beyond the presence of a doublet corresponding to unfolded and folded Laz in WT bacteria, only a very subtle protein band of ∼50 kDa could be observed in the presence of the highest cross-linker concentrations in WT cells when assessed with anti-Laz antiserum, while no signal was observed in the Δ*laz* mutant (Figure 6E). The band around 50 kDa could correspond to an interaction between Laz and other members of the gonococcal electron transport system. For example, the cytochromes CcoO (22.5 kDa), *c*_5_ (28.8 kDa), *c*_4_ (22 kDa), and the Fe–S subunit of cytochrome *c*_1_ (20.6 kDa) are all within the range of molecular masses that could result in a complex of approximately 50 kDa if they interact with Laz. No difference in the AniA cross-linking pattern was observed between WT and Δ*laz* samples, nor were any bands corresponding to the predicted size of an AniA/Laz complex evident in either blot (∼75 kDa).

Two-dimensional nuclear magnetic resonance demonstrated a weak, transient interaction between *N. gonorrhoeae* Laz and Ccp facilitated by hydrophobic protein interfaces (Nobrega and Pauleta, 2018). We did not observe a signal that would correspond to a Laz/Ccp complex during *in vivo* cross-linking (∼65 kDa). Laz, AniA, and Ccp all contain numerous lysine residues, including a patch of three lysines on the surface of Laz, giving this protein a small, positively charged patch on the surface (Nobrega and Pauleta, 2018). Any interactions within the timescale of the DSP reaction should be captured. However, to address the possibility that Laz and AniA interact more transiently than DSP can detect, we employed biolayer interferometry (BLI) using purified Laz immobilized to a BLI sensor and incubated in increasing concentrations of AniA. We have successfully used BLI to interrogate the interaction between AniA and a series of peptide inhibitors (Sikora et al., 2017), as well as the binding of human lysozyme by a surface-exposed gonococcal lysozyme inhibitor, SliC (Zielke et al., 2018). Similar to our cross-linking results, no AniA/Laz interaction was detected by this *in vitro* approach (Supplementary Figure S3).

With the series of experiments presented in Figure 6 and Supplementary Figure S3, we have used comprehensive complementary techniques to test whether Laz and AniA interact. First – and most importantly – we established that Laz and AniA reside in distinct subcellular locations. Second, our studies showed that while AniA is upregulated at later stages of infection of the female murine lower genital tract, Laz abundance is greatly diminished (Figure 6A). AniA is a major anaerobic porin essential for bacterial survival during anoxia (Clark et al., 1987), and its expression is barely detectable under aerobic conditions (Zielke et al., 2014; Figure 6B). There were no changes in AniA levels in anaerobically cultured *Δlaz* gonococci; however, a slight reduction was noted during aerobic growth (Figure 6B). These results indicate that the two proteins are not genetically linked and are not under the same transcriptional control. Third, *in vivo* crosslinking demonstrated that Laz and AniA do not interact within the *N. gonorrhoeae* CE (Figures 6D,E). Finally, BLI using purified proteins revealed no interaction between the two isolated proteins *in vitro* (Supplementary Figure S3).

## Conclusion

Herein, we have shown that despite certain useful attributes such as high conservation and expression in a wide-range of clinical isolates, Laz is not a suitable gonorrhea vaccine candidate due to its periplasmic location. We also performed characterization of Laz function within the *N. gonorrhoeae* cell envelope. We demonstrated for the first time that Laz and AniA are expressed during gonococcal infection in the lower genital tract of female mice, suggesting that different components of the electron transport chain may be active *in vivo*. Importantly, our experiments clarified the literature, which previously suggested Laz as the proteinaceous electron donor to AniA (Boulanger and Murphy, 2002; Nobrega et al., 2016), and its surface exposure in *N. meningitidis* (Deeudom et al., 2015), further highlighting existing differences in homologous protein localization between closely related species (Figure 1; Sikora et al., 2018, BamE; Jongerius et al., 2013, factor H binding protein]. Our studies are also the first to establish the feasibility of crosslinking to capture protein–protein interactions in living gonococci, which could be beneficial for the *Neisseria* research community to map protein–protein interaction networks.

## Author Contributions

AS and BB developed and designed the study, analyzed data, and wrote the manuscript. BB and RZ performed the experiments. AJ provided reagents and assisted with manuscript revisions.

## Conflict of Interest Statement

The authors declare that the research was conducted in the absence of any commercial or financial relationships that could be construed as a potential conflict of interest.

## Footnotes

1 https://pubmlst.org/neisseria/
