# Supplementary material for: Lipid-Modified Azurin of Neisseria gonorrhoeae Is Not Surface Exposed and Does Not Interact With the Nitrite Reductase AniA
Source: Front Microbiol. 2018 Nov 27;9:2915. doi: 10.3389/fmicb.2018.02915 (PMC6277709; doi:10.3389/fmicb.2018.02915)
Supplement: Supplementary file 1 [file Data_Sheet_1.PDF]

Supplementary Material

Lipid-modified azurin of *Neisseria gonorrhoeae* is not surface exposed and does not interact with the nitrite reductase AniA

Benjamin I. Baarda[1], Ryszard A. Zielke[1], Ann E. Jerse[3], and Aleksandra E. Sikora[1,2]*

[1]Department of Pharmaceutical Sciences, College of Pharmacy, Oregon State University, Corvallis, Oregon, United States

[2]Vaccine and Gene Therapy Institute, Oregon Health & Science University, Beaverton, Oregon, United States

[3]Department of Microbiology and Immunology, F. Edward Hebert School of Medicine, Uniformed Services University of the Health Sciences, Bethesda, Maryland, United States

*** Correspondence:**
Aleksandra Sikora
Aleksandra.Sikora@oregonstate.edu

SUPPLEMENTAL MATERIALS AND METHODS

Biolayer interferometry. Interactions between AniA and Laz were evaluated by Biolayer Interferometry. Recombinant Laz and AniA were purified as described (Zielke et al., 2016; Sikora et al., 2017). Purified Laz was biotinylated with Pierce EZ-Link Sulfo-NHS-Biotin reagent (ThermoFisher Scientific) according to manufacturer's instructions. Briefly, protein storage buffer was exchanged to phosphate buffered saline using a PD-10 desalting column (GE Healthcare Life Sciences). Biotin reagent was added to Laz at a 20-fold molar excess and incubated at room temperature for 1 h. Excess biotin was removed by desalting on a PD-10 column. Biotinylated Laz was diluted to 20 µg/mL in kinetic assay buffer (20 mM HEPES pH 8, 15 mM NaCl, 0.002% Tween 20, 0.1 mg/mL BSA) and immobilized to a streptavidin-coated fiber-optic biosensor (ForteBio) at room temperature for 10 minutes. Laz-coated sensors were incubated with indicated concentrations of AniA diluted in kinetic assay buffer using an Octet RED96 system (ForteBio), then transferred to kinetic buffer only to monitor association and dissociation, respectively. Baseline readings were established for 240 s, and association and dissociation steps were performed for 500 s. Empty tips were used as controls. Experiments were performed on two occasions and a representative graph is presented.

REFERENCES:

Sikora, A.E., Mills, R.H., Weber, J.V., Hamza, A., Passow, B.W., Romaine, A., et al. (2017). Peptide Inhibitors Targeting the Neisseria gonorrhoeae Pivotal Anaerobic Respiration Factor AniA. *Antimicrob Agents Chemother* 61(8). doi: 10.1128/AAC.00186-17.

Zielke, R.A., Wierzbicki, I.H., Baarda, B.I., Gafken, P.R., Soge, O.O., Holmes, K.K., et al. (2016). Proteomics-driven Antigen Discovery for Development of Vaccines Against Gonorrhea. *Mol Cell Proteomics* 15(7)**,** 2338-2355. doi: 10.1074/mcp.M116.058800.

Supplemental Figure S1. Phylogenetic relationships between *Neisseria* Laz alleles. Maximum likelihood trees, constructed in MEGA7 with the Jones-Taylor-Thornton method, were generated for all Laz alleles found across Neisseria alleles found in the PubMLST Neisseria database. FA1090 Laz allele is boxed in red (allele 15).

Supplemental Figure S2. Microscopic examination of exposure to 16 mM nitrite. Rapidly growing liquid cultures of indicated strains were standardized to an OD_{600} of 0.2, serially diluted, and spotted onto GCB supplemented with 0.1 mM IPTG and either without or with 16 mM $NaNO_2$. Spots were visualized with a Zeiss AxioObserver.D1 at 2.5× magnification 0.06 Phase Contrast 1 at the -4 dilution for the 0 $NaNO_2$ condition (A), and at 10× magnification 0.25 Phase Contrast 1 at the -1 dilution for the 16 mM $NaNO_2$ condition (B). Each spot’s border is denoted by an arrow in (B).

Supplemental Figure S3. Biolayer Interferometry between Laz and AniA. Biotinylated Laz was immobilized to streptavidin-coated fiber optic biosensors and incubated with indicated concentrations of AniA to monitor for association. Sensors were subsequently incubated in buffer without AniA to monitor for dissociation.

Figure S1

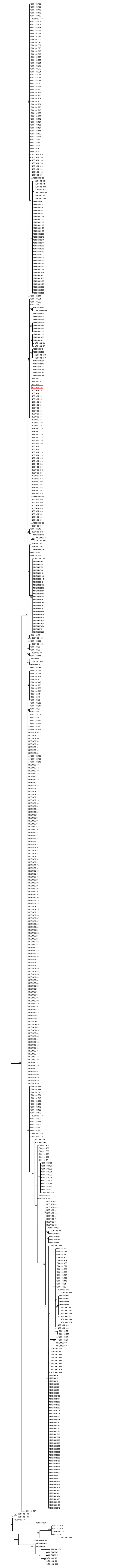

Supplemental Figure S1. Phylogenetic relationships between *Neisseria* Laz alleles. A maximum likelihood tree, constructed in MEGA7 with the Jones-Taylor-Thornton method, was generated for all Laz alleles found across *Neisseria* species found in the PubMLST *Neisseria* database. FA1090 Laz allele is boxed in red (allele 15).

Figure S2

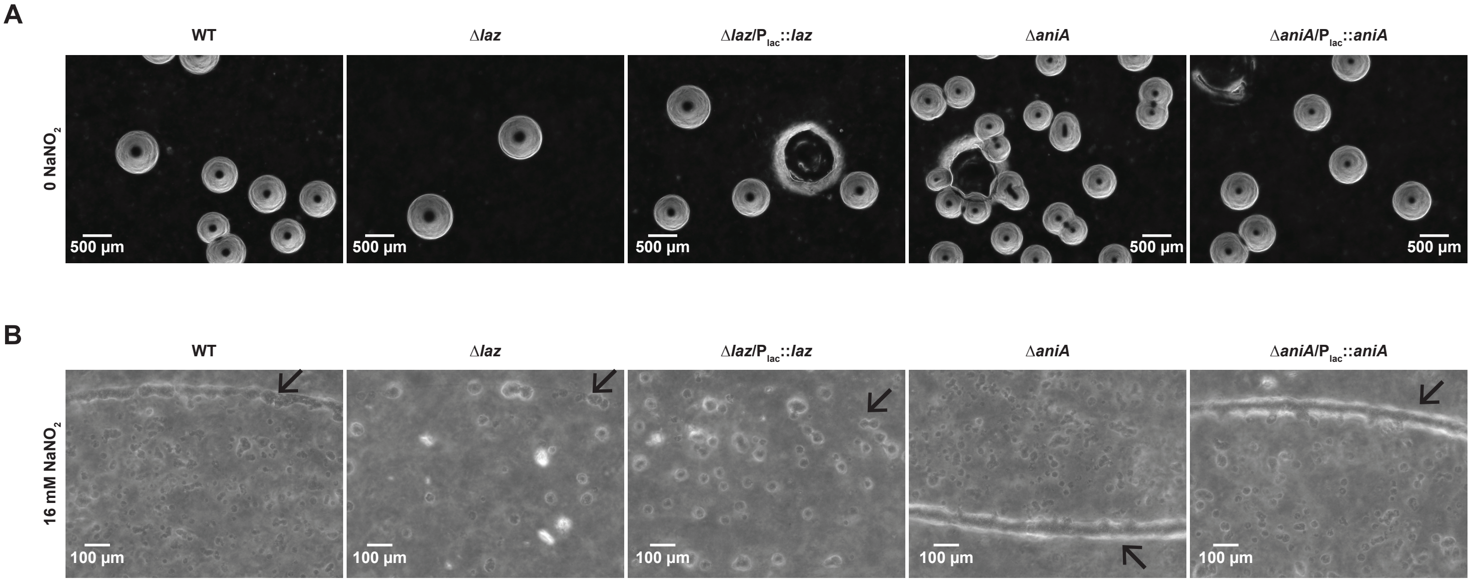

Supplemental Figure S2. Microscopic examination of exposure to 16 mM nitrite. Rapidly growing liquid cultures of indicated strains were standardized to an OD_{600} of 0.2, serially diluted, and spotted onto GCB supplemented with 0.1 mM IPTG and either without or with 16 mM $NaNO_2$. Spots were visualized with a Zeiss AxioObserver.D1 at 2.5× magnification 0.06 Phase Contrast 1 at the -4 dilution for the 0 $NaNO_2$ condition (A), and at 10× magnification 0.25 Phase Contrast 1 at the -1 dilution for the 16 mM $NaNO_2$ condition (B). Each spot's border is denoted by an arrow in (B).

Figure S3

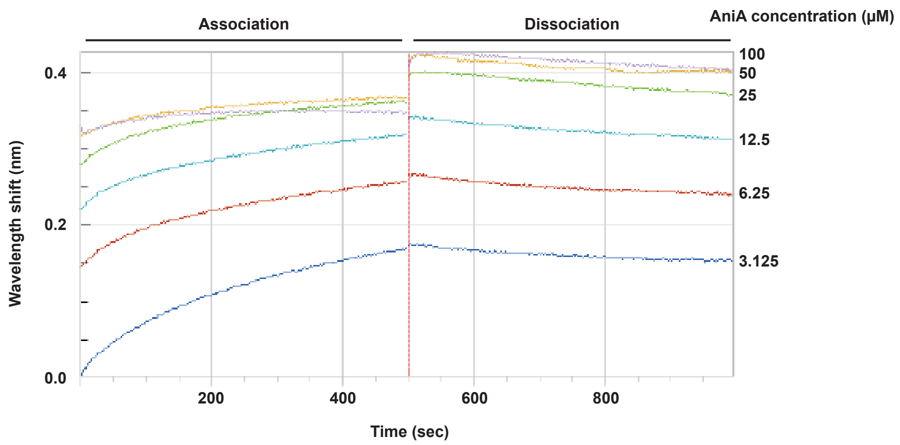

Supplemental Figure S3. Biolayer Interferometry between Laz and AniA. Biotinylated Laz was immobilized to streptavidin-coated fiber optic biosensors and incubated with indicated concentrations of AniA to monitor for association. Sensors were subsequently incubated in buffer without AniA to monitor for dissociation.
